# Supplementary figures and images for: Step-by-Step Regeneration of Tentacles after Injury in Anemonia viridis—Morphological and Structural Cell Analyses
Source: Int J Mol Sci. 2023 May 16;24(10):8860. doi: 10.3390/ijms24108860 (PMC10219038; doi:10.3390/ijms24108860)

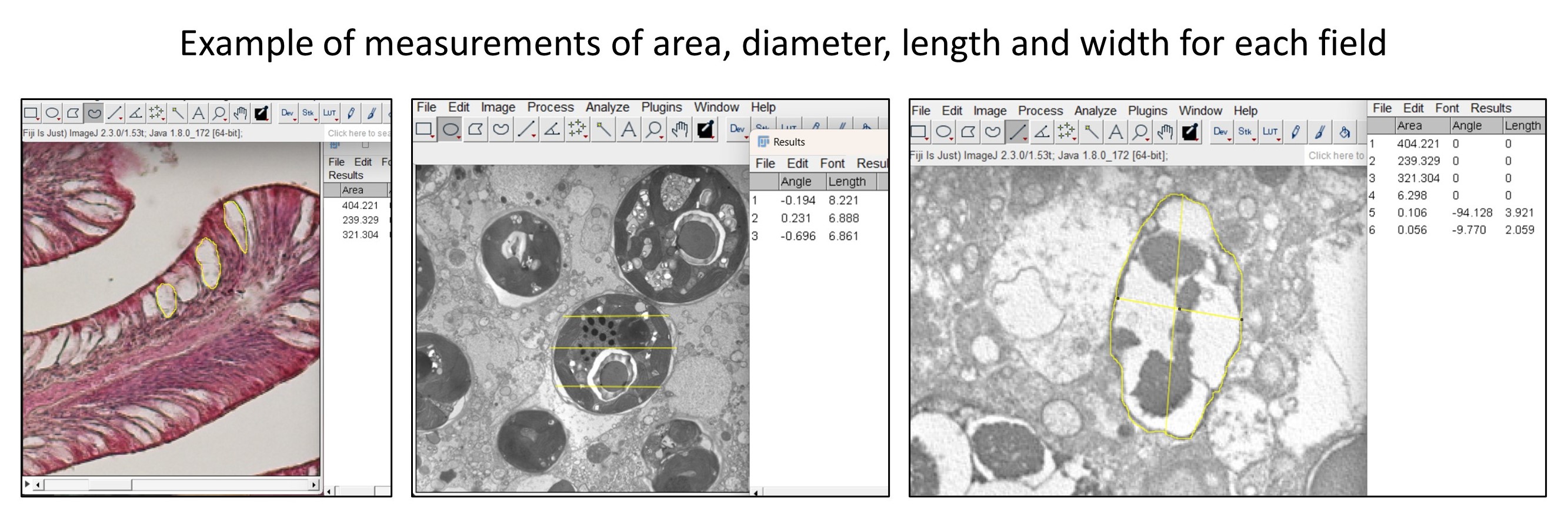

Supplement: Supplementary file 1 [file ijms-24-08860-s001.zip › ijms-2354840-supplementary.jpg]
